# Sex Differences in Lung Cancer Characteristics at Diagnosis: A Nationwide Register‐Based Study From the Faroe Islands (2015–2020)

**DOI:** 10.1002/cnr2.70066

**Published:** 2025-01-23

**Authors:** Annika H. Lindenskov, Halla Potts, Marnar F. Kristiansen, Maria S. Petersen, Marin Strøm

**Affiliations:** ^1^ Centre of Health Science University of the Faroe Islands Tórshavn Faroe Islands; ^2^ The Faroese Cancer Registry Tórshavn Faroe Islands; ^3^ National Hospital of the Faroe Islands Tórshavn Faroe Islands; ^4^ Department of Research The National Hospital of the Faroe Islands Tórshavn Faroe Islands

**Keywords:** epidemiology, Faroe Islands, lung cancer, sex differences, survival rates

## Abstract

**Background:**

Sex differences in lung cancer survival are well‐established, but the gap between Faroese men and women is especially pronounced. Faroese women have some of the highest 1‐ and 5‐year relative survival rates in the Nordic region, while Faroese men have some of the lowest. This study investigates these survival disparities by analyzing demographic, clinical, and temporal factors in Faroese lung cancer patients from 2015 to 2020.

**Methods:**

All lung cancer cases registered in the Faroese Cancer Registry from 2015 to 2020 were included. Data on age, sex, civil status, cancer type, stage, smoking history, comorbidities, and performance status were extracted from electronic patient records. Sex‐based differences were analyzed and overall survival rates were estimated using Kaplan–Meier plots.

**Results:**

Significant sex differences were observed in cancer staging at diagnosis (*p* = 0.03), with 25.8% of women diagnosed at Stage I compared to 8.8% of men. Gender‐specific patterns also emerged: women hadlonger symptomatic periods, while men experienced longer diagnostic and treatment times, though with overlapping confidence intervals.

**Conclusions:**

Our findings reveal significant sex disparities in lung cancer staging at diagnosis in the Faroe Islands, which may the survival differences. The longer diagnostic period in men likely contributes to their lower survival rates. These results highlight the need for targeted interventions to reduce these disparities and improve patient outcomes.

## 1 Introduction

Lung cancer is the leading cause of cancer‐related deaths in both men and women worldwide [1, 2, 3], including in the geographically isolated Faroe Islands [4]. The Faroe Islands, with a population of 54 908 inhabitants [5], are a welfare state and a small‐island society where familism serves as a key social and cultural pillar [6, 7, 8]. The Faroese healthcare system is tax‐funded, offering free access to general practice (GP), outpatient, and hospital care. While the system is modern, local specialized medical care is limited due to the small population size, necessitating cooperation with other countries. For example, lung cancer patients undergo surgery and radiotherapy in Denmark.

Cancer incidence rates in the Nordic countries are regularly reported through NORDCAN, and over the past two decades, lung cancer incidence has declined in these countries. However, in the Faroe Islands, there has been a slight increase in lung cancer incidence during the same period [9]. The NORDCAN database also provides cancer survival statistics for the Nordic countries. For lung cancer, men consistently exhibit lower relative survival compared to women, and this trend is also observed in the Faroe Islands. Faroese women have relatively high 1‐ and 5‐year survival (59.7% and 31.6%, respectively), whereas men have notably lower 1‐ and 5‐year survival (43.4% and 12.7%, respectively) [10, 11, 12].

In this study we investigate whether factors such as age, smoking habits, clinical characteristics, and patient timelines among Faroese lung cancer patients between 2015 and 2020 differ by sex, and thus may explain the observed survival disparity between male and female lung cancer patients in the Faroe Islands.

## 2 Material and Methods

The Faroese Cancer Register (FCR) is a nationwide, population‐based registry that records all incident cases of cancer and cancer‐related mortality and provides data to NORDCAN. In this study, we extracted data on personal identity number (PIN), date of birth, sex, age, diagnosis (ICD code), morphology, and cause of death (ICD code). After excluding one duplicate registration, the total number of cases included in this study was 153. Additional variables were obtained by linking the FCR data to electronic medical records using the PIN, including civil status, smoking history, comorbidities, surgery, Eastern Cooperative Oncology Group performance status (ECOG PS, a measure of daily living activities) [13], lung cancer type, and stage. If multiple records were available, the one closest to the date of diagnosis was used. In cases where the cancer stage was not recorded in the electronic medical record, we determined the stage based on histology reports or computed tomography (CT) scan descriptions, following the TNM classification guidelines from the American Joint Committee and Union Internationale Contre le Cancer [14].

### 2.1 Definition of Timeline

The timelines were derived from hospital electronic medical records and the GP referrals for x‐rays, as direct access to GP medical records was not available. The timelines represent three phases in the patient pathway:

- The *symptomatic period*: the number of days from the first reported symptom to the date the GP sent the hospital referral.
- The *diagnostic period*: the number of days from the GP's referral to the hospital until the date of diagnosis.
- The *time to treatment period*: the number of days from the date of diagnosis to the start of treatment.

### 2.2 Ethics

This study complied with the Faroese Data Protection Act and was conducted under the supervision of the Data Protection Officer of the Faroese Hospital Service. As a registry‐based project without the use of biological samples, the study was exempt from approval by the Scientific Ethical Committee in accordance with Faroese legislation [15].

### 2.3 Statistical Analysis

Statistical analyses were conducted using the IBM SPSS (version 29.0; SPSS Inc. Chicago, Il, USA). Clinical characteristics were presented as *n* (%) for categorical variables, and the Pearson chi‐squared test was used to assess sex differences. For the continuous variable, age, the mean (standard deviation (SD)) was reported, and the Student's *t*‐test was applied after evaluating normality using visual plot inspection and the Kolmogorov–Smirnov test. Age was dichotomized into ≤ 70 years and > 70 years, and cancer stage was categorized as low (Stage I/II) or high (Stage III/IV).

Overall survival was analyzed using the Kaplan–Meier method, with the log rank test employed to compare survival between groups. Study participants were followed from the date of diagnosis until death, or the end of follow‐up (20.11.22), whichever occurred first. Timelines were calculated in Excel (version 2022), and each period was reported as the mean number of days with 95% confidence intervals (CI). A *p*‐value < 0,05 was considered statistically significant.

## 3 Results

The majority of the 153 patients diagnosed with lung cancer in the Faroe Islands from 2015 to 2020 were men (60%, Table 1). The mean age at diagnosis was 70 years (SD 10.1) and a higher proportion of men (57%) than women (48%) were older than 70 years at the time of diagnosis, though this difference was not statistically significant. A significant sex difference was observed in cancer stage at diagnosis (*p* = 0.03), particularly in Stage I, where 26% of women and only 9% of men were diagnosed. The majority of both men and women were diagnosed at Stage IV (Table 1).

**TABLE 1:** Clinical characteristics of men and women with lung cancer, Faroe Islands, 2015–2020, *n* = 153.

		Men	Women	
		*n* = 91 (59.5%)	*n* = 62 (40.5%)	
Variable	Category	*n*%	*n*%	*p*
Age	≤ 70	39 (42.9)	32 (51.6)	0.286^a^
	≻ 70	52 (57.1)	30 (48.4)	
Civil status	Married	56 (61.5)	34 (54.8)	0.264^a^
	Not married	29 (31.9)	19 (30.6)	
	Unknown	6 (6.6)	9 (14.5)	
Smoking	Smokers	48 (52.7)	24 (38.7)	0.259^a^
	Former smoker	38 (41.8)	31 (50.0)	
	Never smoker	3 (3.3)	3 (4.8)	
	Unknown	2 (2.2)	4 (6.5)	
ECOG PS^b^	0–1	49 (53.8)	34 (54.8)	0.434^a^
	2	22 (24.2)	11 (17.7)	
	3–4	19 (20.9)	14 (22.6)	
	Unknown	1 (1.1)	3 (4.8)	
Lung cancer type	SCLC^c^	16 (17.6)	13 (21.0)	0.710^a^
	NSCLC^d^	68 (74.7)	46 (74.2)	
	Unknown	7 (7.7%)	3 (4.8)	
Stage	I	8 (8.8)	16 (25.8)	0.031^a^
	II	12 (13.2)	5 (8.1)	
	III	29 (31.9)	14 (22.6)	
	IV	42 (46.2)	27 (43.5)	
Stage group	Low (Stage I + II)	20 (22.0)	21 (33.9)	0.103^a^
	High (Stage III + IV)	71 (78.0)	41 (66.1)	
Surgery	Surgery	20 (22.0)	15 (24.2)	0.708^a^
	No surgery	71 (78.0)	46 (74.2)	
Comorbidity	Yes	76 (83.5)	52 (83.9)	0.478^a^
	No	15 (16.5)	10 (16.1)	

^a^ Pearson Chi‐Square test.

^b^ Eastern Cooperative Oncology Group Performance status at diagnosis.

^c^ Small cell lung cancer.

^d^ Non‐small cell lung cancer.

Most patients, both men and women, were diagnosed with non‐small cell lung cancer (NSCLC), had at least one comorbidity, and had an ECOG PS of 0–1. Women underwent surgery slightly more often than men, but this difference was not statistically significant. Patients who did not undergo surgery received chemotherapy, radiotherapy, immunotherapy, a combination of these treatments, or did not receive any treatment (data not shown). Most lung cancer patients were classified as either current or former smokers. Although not statistically significant, more men were active smokers, whereas more women were former smokers.

**FIGURE 1:**
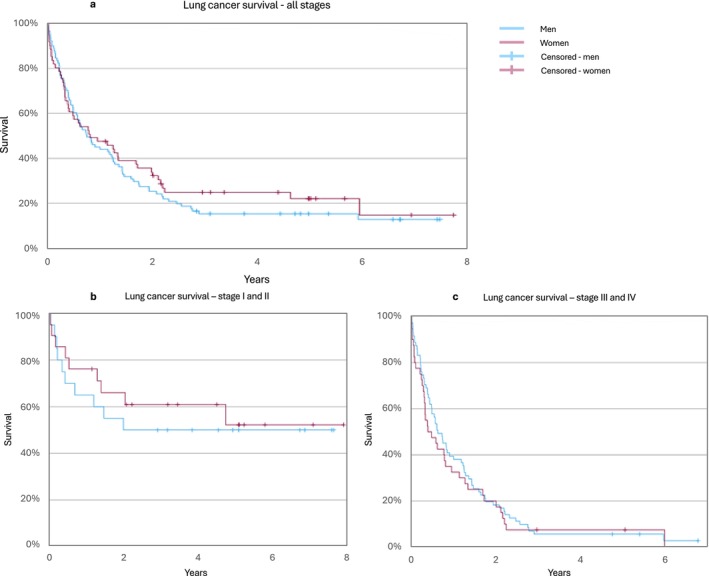
Kaplan–Meier plot showing overall survival for men and women with lung cancer (1a), overall survival for men and women with lung cancer diagnosed at low stage (Stage I and II) (1b), overall survival for men and women with lung cancer diagnosed at high stage (Stage III and IV) (1c).

Overall, no statistically significant differences in survival were observed over the entire study period, as estimated by the Kaplan–Meier survival curves (Figure 1). However, there was a trend toward improved survival for women compared to men during the first 4 years of follow‐up, particularly for those diagnosed at an early stage.

### 3.1 Timelines in Patient Pathways

**FIGURE 2:**
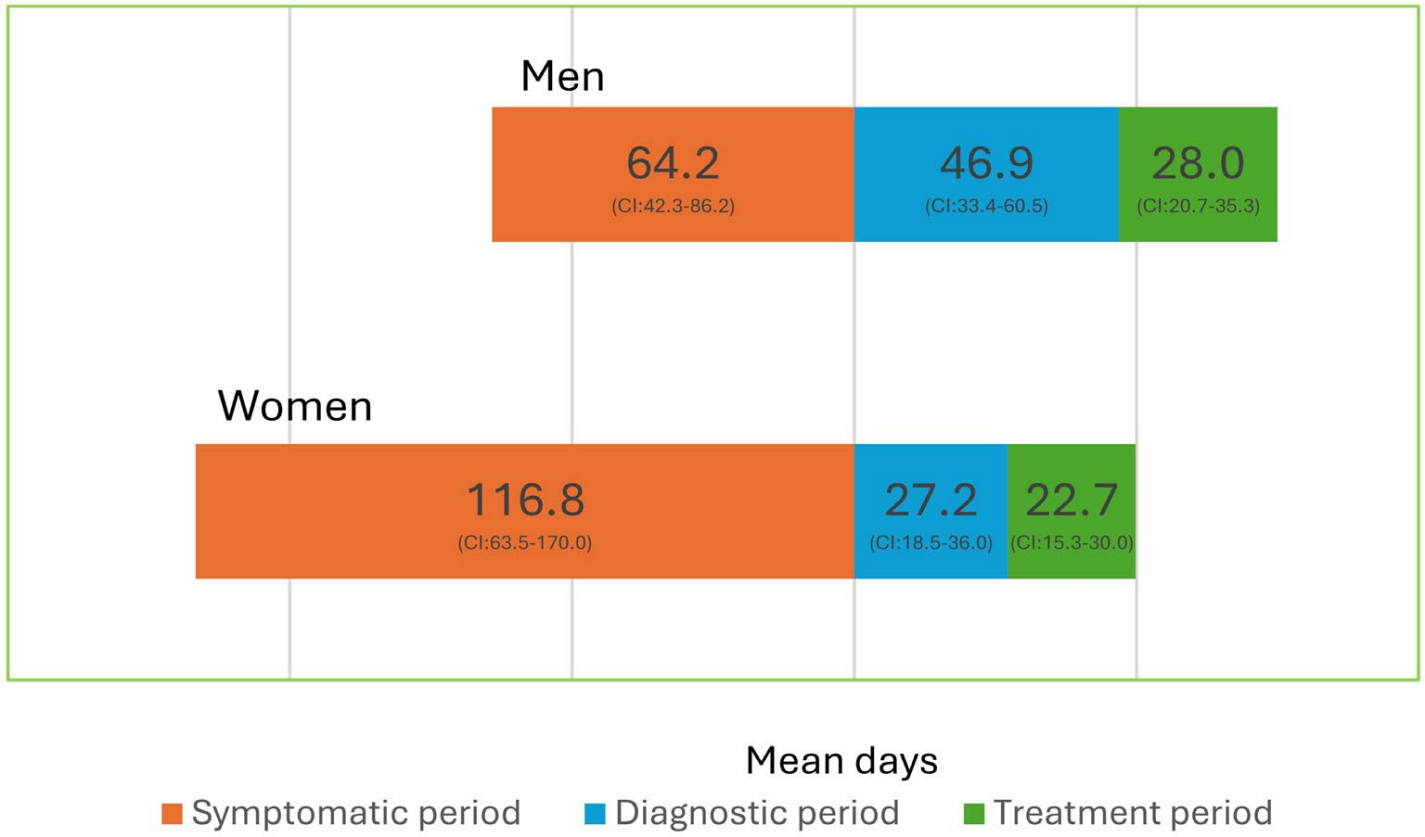
Timeline in mean days for Faroese men and women with lung cancer diagnosed from 2015 to 2020.

For men (*n* = 72), the symptomatic period lasted an average of 64.2 days (range 0–491, 95% CI: 42.2–86.2) compared to 116.8 days (range 0–910, 95% CI: 63.5–170.0) for women (*n* = 45) (Figure 2), a difference of 52.6 days. However, the confidence intervals overlapped. The diagnostic period was 19.7 days longer for men (*n* = 91), averaging 46.9 days (range 0–412, 95% CI: 33.4–60.5) compared to 27.2 days (0–218, 95% CI: 18.5–36.0) for women (*n* = 62), with slight overlap in the confidence intervals. The time to treatment period averaged 28.0 days (0–166, 95% CI: 20.7–35.3) for men (*n* = 59) and 22.7 days (range 0–110, 95 CI: 15.3–30.0) for women (*n* = 41), a difference of 5.4 days, again with overlapping confidence intervals.

## 4 Discussion

We identified a sex difference in the stage at diagnosis of lung cancer in the Faroe Islands, consistent with other Nordic studies showing a survival advantage for women, even when diagnosed at the same stage as men [16, 17, 18]. Early‐stage diagnosis offers the potential for surgical treatment [2]. In our study, women were more likely than men to undergo surgery, although this difference was not statistically significant. This pattern aligns with observations in Danish patient populations [17], and a Swedish study also demonstrated that women had better survival than men, even among those who underwent surgery [19]. Similarly, a Norwegian study found poorer survival outcomes for men compared to women, possibly due to lower rates of surgical treatment, later stage at diagnosis, higher smoking rates, more comorbidities, and worse ECOG PS among men [20]. In our study, comorbidity and ECOG PS were similar between the sexes, but more men were diagnosed at a late stage and were active smokers, even though these differences were not statistically significant.

In lung cancer progression, time is critical, and a shorter symptomatic period may be associated with a poorer prognosis, as patients may present with symptoms at a more advanced stage [21]. Therefore, rapid treatment initiation is expected for patients diagnosed at a late stage [21]. Although not statistically significant, the symptomatic period was shorter for men compared to women in our study, whereas both the diagnostic and time‐to‐treatment periods showed a sex disparity favoring women. These delays may negatively affect men's survival.

Sex differences in lung cancer survival may be due to biological or sociocultural factors. More aggressive tumor behavior has been observed in men [18, 19], likely contributing to the lower survival seen among Faroese men. However, the etiology is likely multifactorial. Sociocultural norms may also play a role. Traditional masculinity is often associated with riskier behaviors, including reluctance to seek medical care [18, 22, 23, 24, 25], and illness may evoke feelings of inadequacy or vulnerability, especially regarding the family's support [8, 22, 26]. Furthermore, women may respond more proactively to symptoms than men [24, 27]. Since traditional gender roles remain relatively strong in the Faroe Islands [19, 24], these factors could help explain our findings.

The strengths of our study include its nationwide, population‐based design, the completeness of case data, and the detailed review of individual patient electronic medical records. However, the small sample size (*n* = 153) limits the study's statistical power, increasing the risk of type II errors. Clinical stage at diagnosis was not reported for 45% of patients; for these cases, we classified stage based on histology and CT descriptions using the TNM classification system [14, 28], which could introduce bias through misclassification. However, we believe this is unlikely to have significantly impacted our findings, as the majority (75%) of these patients were classified at an advanced stage, and there is evidence that the risk of misclassification bias is lower for patients at later stages [17].

In the timelines, we calculated days as a mean rather than a median. Although outliers can have a strong impact on the mean, it allows for summing the periods and comparing the timelines with those of cancer patients in other countries.

The timelines shown in this study have some limitations. The retrospective self‐reported data on symptom onset is subject to recall bias. Due to the difficulty in determining the exact date of first symptoms [29], the symptomatic period was based on 115 patients out of the full sample of 153. The treatment period was based on data from 100 patients, primarily due to missing information about the date of first treatment which often occurs in Denmark. The diagnostic period was constructed from the entire study population and is therefore less prone to information or selection bias.

In the Nordic countries, cancer survival has improved following the implementation of cancer patient pathways [10, 11, 18]. Such pathways have not yet been implemented in the Faroe Islands. However, in 2018, a seven‐day timeframe from referral on suspicion of cancer to consultation with a pulmonary physician was introduced [27]. Our study includes patients diagnosed up to 2020, meaning the full impact of this timeframe may not yet be apparent. An ad‐hoc analysis comparing timelines before and after introduction the guideline's introduction did not show any reduction in the observed sex differences in timelines. Following the example of the Nordic countries, the introduction of formal cancer patient pathways is expected to improve survival outcomes for lung cancer patients in the Faroe Islands and may help reduce the observed sex disparities.

## 5 Conclusion

In this study, which included all Faroese lung cancer patients from 2015 to 2020, we found that men were diagnosed at a more advanced stage than women, and that patient timelines varied between the sexes, although these differences were not statistically significant. Our results suggest a delay in diagnostic and treatment periods for men, potentially contributing to their markedly lower survival. Implementing national lung cancer pathways in the Faroe Islands would be key to ensuring systematic and equitable care for all lung cancer patients and is likely to reduce sex disparities and improve overall cancer outcomes.

## Author Contributions

**Annika H. Lindenskov:** writing – original draft, funding acquisition, methodology, formal analysis, conceptualization, validation, project administration, investigation, writing – review and editing. **Halla Potts:** conceptualization, funding acquisition, writing – original draft, methodology, validation, formal analysis, writing – review and editing, project administration, investigation. **Marnar F. Kristiansen:** data curation, writing – review and editing, writing – original draft, supervision, validation, methodology. **Maria S. Petersen:** writing – review and editing, supervision, data curation, writing – original draft. **Marin Strøm:** writing – review and editing, writing – original draft, supervision, data curation, methodology.

## Disclosure

The authors alone are responsible for the content and writing of this article.

## Conflicts of Interest

The authors declare no conflicts of interest.

## Data Availability

The data that support the findings of this study are available from the corresponding author upon reasonable request.
